# Three-dimensional data capture and analysis of intact eye lenses evidences emmetropia-associated changes in epithelial cell organization

**DOI:** 10.1038/s41598-020-73625-9

**Published:** 2020-10-09

**Authors:** Alexia A. Kalligeraki, Archie Isted, Miguel Jarrin, Alice Uwineza, Robert Pal, Christopher D. Saunter, John M. Girkin, Boguslaw Obara, Roy A. Quinlan

**Affiliations:** 1 Department of Biosciences, Durham University, South Road Science Site, Durham, DH1 3LE UK; 2 Department of Chemistry, Durham University, South Road Science Site, Durham, DH1 3LE UK; 3 Department of Physics, Durham University, South Road Science Site, Durham, DH1 3LE UK; 4 Department of Computer Science, Durham University, South Road Science Site, Durham, DH1 3LE UK

**Keywords:** Image processing, Software, Organogenesis, Visual system, Intermediate filaments

## Abstract

Organ and tissue development are highly coordinated processes; lens growth and functional integration into the eye (emmetropia) is a robust example. An epithelial monolayer covers the anterior hemisphere of the lens, and its organization is the key to lens formation and its optical properties throughout all life stages. To better understand how the epithelium supports lens function, we have developed a novel whole tissue imaging system using conventional confocal light microscopy and a specialized analysis software to produce three-dimensional maps for the epithelium of intact mouse lenses. The open source software package geometrically determines the anterior pole position, the equatorial diameter, and three-dimensional coordinates for each detected cell in the epithelium. The user-friendly cell maps, which retain global lens geometry, allow us to document age-dependent changes in the C57/BL6J mouse lens cell distribution characteristics. We evidence changes in epithelial cell density and distribution in C57/BL6J mice during the establishment of emmetropia between postnatal weeks 4–6. These epithelial changes accompany a previously unknown spheroid to lentoid shape transition of the lens as detected by our analyses. When combined with key findings from previous mouse genetic and cell biological studies, we suggest a cytoskeleton-based mechanism likely underpins these observations.

## Introduction

The lens is an avascular tissue located in the anterior chamber of the eye that grows throughout the life of the individual. Its physiological role is to refract light onto the retina and this function must be maintained initially as the eye develops, but then adapt to different growth requirements as the eye ages^1–4^. For mammals, the postnatal period of development is where the most accelerated growth phase takes place and when the growth of the different tissues of the eye is integrated to deliver the required visual properties^5^. These vary between mammals and are related to their environment and behaviors, for example whether they are nocturnal or aquatic^4,6^. Changes in the eye lens during the early postnatal period are key to understanding these differences^7^. There is, therefore, the need to measure the biometric parameters of the lens^8–10^ and particularly the number, distribution and geometric organization of the lens cells themselves^11^ in order to dissect the molecular pathways that are critical to vision development^12^ and to understand eye conditions such as myopia and cataract^13^.

Lens cells can be divided into two distinct populations. Lens epithelial cells (LECs) form a cuboidal monolayer on the anterior half of the tissue, which differentiate into lens fiber cells (LFCs) at the lens equator, where they also internalize and continually add to the lens mass throughout life^14^. LFCs are the most abundant lens cells and form the bulk of the lens. LFCs become organelle-free as a key part of their differentiation process and are characterized by a high crystallin protein content^15,16^ and regular hexagonal organization^17^.

**Figure 1:**
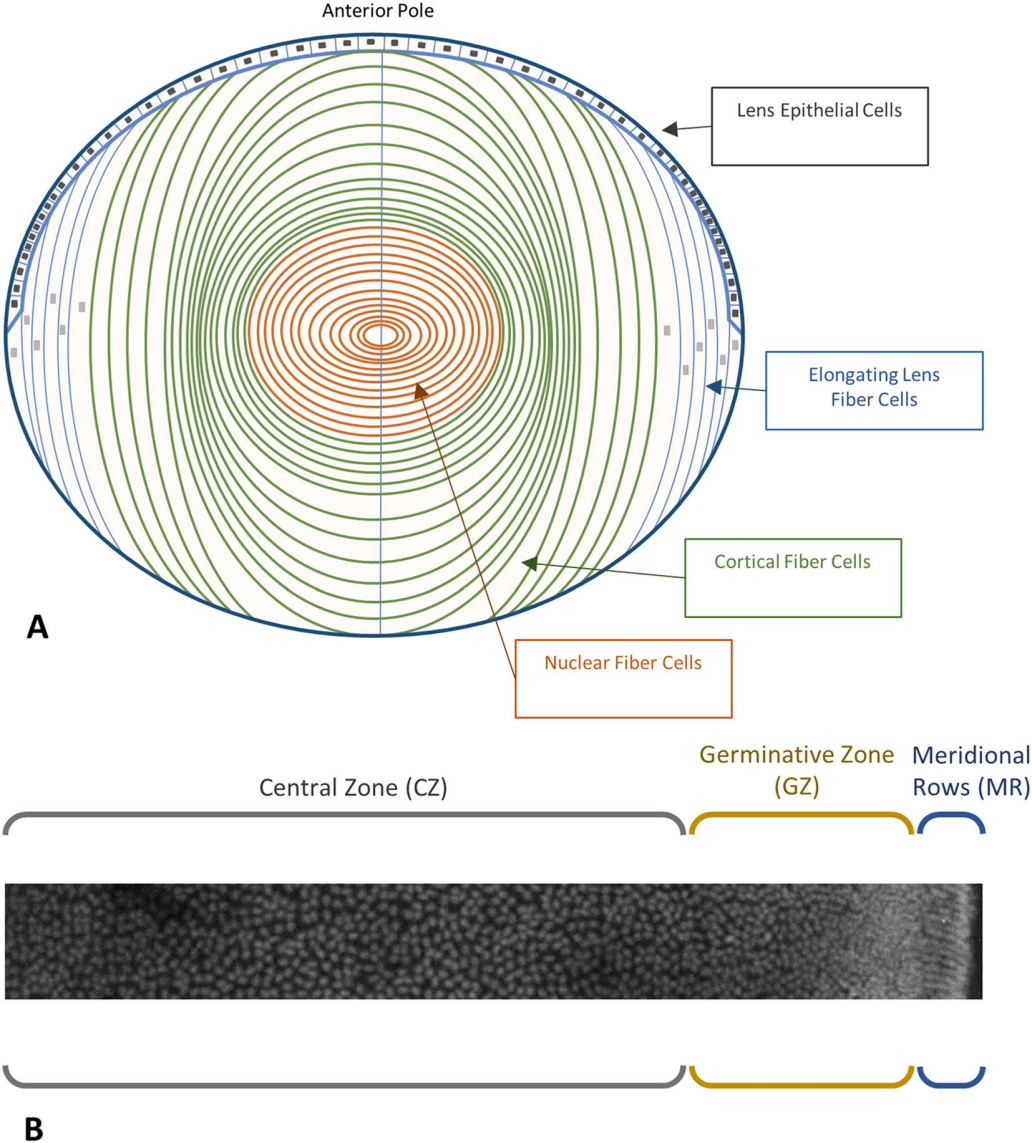
Lens organization and cell distribution in the epithelium. (**A**) Schematic representation of a mammalian lens. (**B**) 3D maximum intensity projection of a murine lens epithelium from anterior pole (left) to meridional rows (right). A coronal section of the lens reveals its major constituents, the lens epithelial cells (LECs), and the lens fiber cells (LFCs). LECs occupy a monolayer on the anterior portion of the lens, whereas LFCs form the bulk of the tissue. LFCs are derived from LECs in the MR and shed their organelles while elongating during their differentiation and maturation. LECs are organized into different zones on the epithelium, with a low-density population (grey bracket) forming the central zone (CZ), a high density region containing actively replicating cells (yellow bracket) for the germinative zone (GZ), and a low-density region of aligned cells (blue bracket) in the meridional rows (MR).

The lens epithelium arguably holds the key to understanding lens formation, growth, and maintenance throughout life. Three morphologically distinct zones have been identified for the lens epithelium, as seen in Fig. 1. The zone covering the anterior pole is termed the central zone (CZ); here cells are largely in a quiescent state. The germinative zone (GZ) lies closer to the lens equator and is where epithelial cells proliferate and begin to transition into differentiating fiber cells. Below the lens equator, these presumptive fiber cells align and form meridional rows (MR), a zone where the first steps of cell elongation and differentiation takes place. As LECs are the reservoir of all future fiber cells, it is important to know how LEC organization changes during lens ageing and also at key stages of growth, especially those that potentially determine the functional contribution of the lens to vision^7^. Mathematical models of the lens epithelium have been produced^11,18^, but they have used methodologies that could introduce inaccuracies into the measurements, particularly of the small, round lenses of mice, rats and zebrafish.

Consequently, there is a need to be able to image the intact lens and to produce a three-dimensional map of the lens epithelium. Calculating the position of the anterior pole and measuring the maximum diameter of the lens equator as well as the lens curvature through computational means aims to minimize inherent user bias. Every LEC can then be identified in terms of Cartesian coordinates and the different cell zones in the epithelium (CZ, GZ, MR) can be characterized on the basis of cell density changes and their Cartesian distribution. This is especially important for the future understanding of how growth factors, such as Fibroblast Growth Factor (FGF) determine gene expression to support fiber cell differentiation^19^ and integrate transcriptional signals to effect changes in lens size and lens epithelial organization^20^. Geometric mapping also facilitates the future analysis of the role played by the eye lens during the development and establishment of emmetropia for the visual apparatus using the mouse as a model system^21^ and the feedback between the different tissues in the eye under different lighting conditions^5,22^, given that the lens is thought to be the key to understanding these and the species differences^7,21^.

## Results

### LEC zonal density decays with age

**Figure 2:**
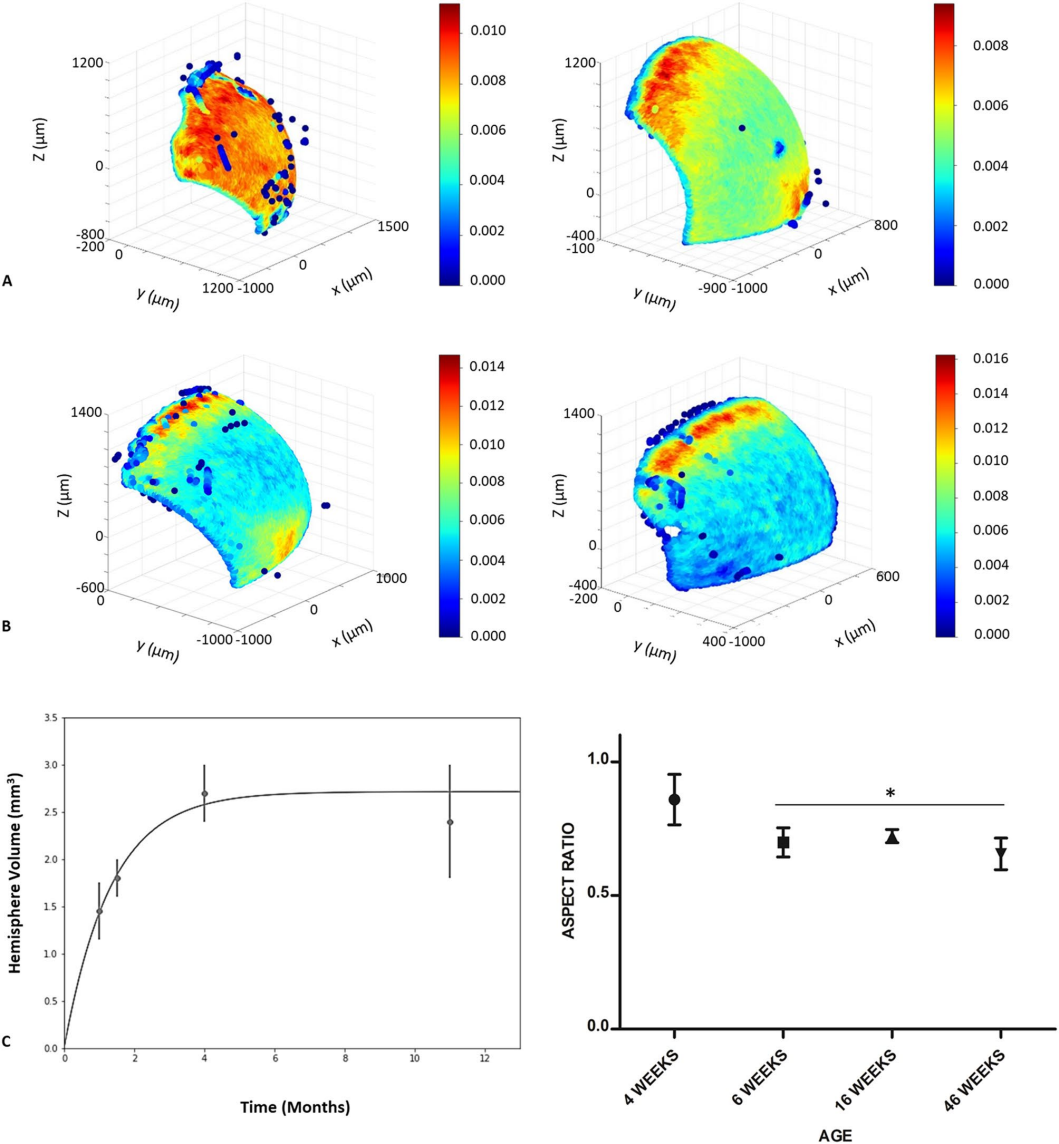
Age dependent changes in cell density, volume, and aspect ratio for the mouse lens. (**A**) C57/BL6J lenses, postnatal weeks 4 (left panel) and 6 (right panel). LEC nuclei density distribution presented as a heat map reconstruction in a five color blue to red scale. At 4 weeks postnatal, cell density is uniform across the epithelium. By 6 weeks, the density gradient from CZ to GZ has developed, though relative high density is retained in the central epithelium zone, possibly to accommodate further lens growth. (**B**) Murine lenses, postnatal weeks 16 (left panel) and 46 (right panel). LEC nuclei density distribution presented as in (**A**). The density gradient has matured by 16 weeks post-natal. (**C**) Murine lens volume and lens aspect ratio (Rmin/Rmax) from 4 to 46 weeks. An initial rapid growth phase during the first 2 months of life is followed by a relative plateau when mice reach maturity. Lens shape on the transverse plane undergoes a spheroid to lentoid transition between postnatal weeks 4 and 6. This lentoid shape is maintained throughout life, with the transverse plane aspect ratio decreasing from 0.9 to 0.7 between postnatal weeks 4 and 6. As hemisphere volume is negatively correlated with aspect ratio, the lens minor axis increases at a greater rate than the major axis. One-way ANOVA with p = 0.0225 for *, standard error bars, n = 16 (4 per age group). Post hoc analysis with Tukey HSD showed a significant decrease in aspect ratio between 4 week lenses and all other age groups (MD = 0.2563 for 6 weeks, MD = 0.2364 for 16 weeks, and MD = 0.2534 for 46 weeks).

Murine lenses develop as a spheroid, uniform high LEC density tissue and retain that cell distribution as seen for the 4-week-old animals. By 6 weeks, lenses have assumed the typical density profile of a low, uniform distribution in the quiescent CZ, a relative high-density population in the GZ, followed by a drop in density and alignment of cells in the MR (Fig. 2A). Cell density in the CZ decays as the tissue ages (cf panels in Fig. 2A,B) after reaching a maximum volume at 16 weeks (Fig. 2C).

### The separation of the high density LEC zone from the CZ at maturation is concurrent with the transition from spheroid to lentoid shape between 4 and 6 weeks

The transition from spheroid to lentoid shape occurs between 4 and 6 weeks postnatal in mice (Fig. 2C). This event is concurrent with the change from uniform high density LEC phenotype to the mature high density GZ and low density CZ phenotype observed in adult mice (Fig. 2A). Still, the relatively high LEC density accommodates rapid tissue growth which is sustained until 16 weeks postnatal (Fig. 2C). Our data show that lens volume is negatively correlated with aspect ratio, as such it is evident that the minor axis increases at a greater rate than the major axis during this time and accounts for this shape transition.

### The maximum density limit of LECs remains adjacent to the equator

**Figure 3:**
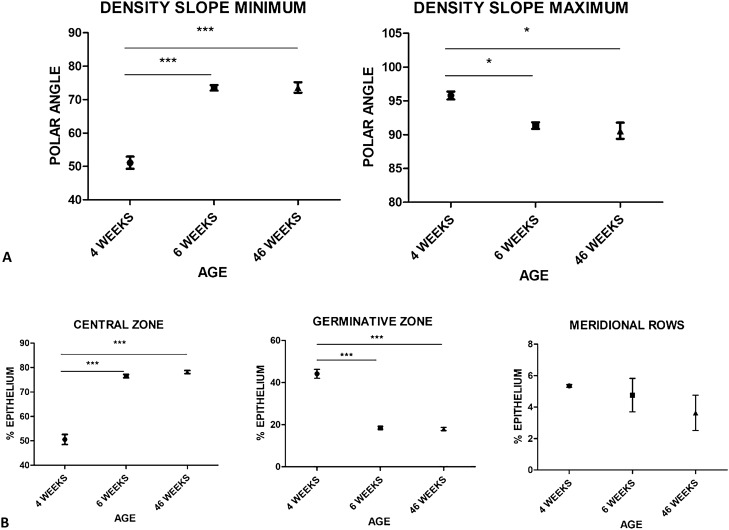
Changes in cell distribution across the lens epithelium with age. (**A**) A density slope minimum of *θ* = 75° remained constant from postnatal week 6 to week 46. One-way ANOVA with p = 0.0128 for * and p = 0.0001 for ***, standard error bars, n = 12 (4 per age group). Post hoc comparisons using the Tukey HSD test showed the mean score was significantly different for the density slope minimum of 6 and 46 week old mice as compared to 4 week old mice (MD = 22.41 for 6 weeks and MD = 22.55 for 46 weeks), but no significant difference was observed between 6 and 46 week old mice (MD = 0.14). Similarly, the density slope maximum of *θ* = 90° was not significantly different between 6 and 46 week old mice (MD = − 0.77;). However, both were significantly different to 4 week old (MD = − 4.45 for 6 weeks and MD = − 5.22 for 46 weeks), where *θ* = 95°. Therefore, a small but significant relative transition of the density slope maximum occurs between weeks 4 and 6. (**B**) LEC zone distribution at postnatal weeks 4, 6, and 46. The contraction of the high density LEC zone occurs between weeks 4 and 6. LEC density is retained through to week 46, with a slight increase in variance observed in increasing age. One-way ANOVA with p < 0.05 for * and p < 0.01 for ***, standard deviation error bars, n = 12 (4 per age group). Post hoc comparisons using the Tukey HSD test showed the mean score was significantly different for the angle range of the CZ of 6 and 46 week old mice as compared to 4 week mice (MD = 25.96 for 6 weeks and MD = 27.62 for 46 weeks), but no statistically significant difference was observed between 6 and 46 week old mice (MD = 1.661). Similarly, the angle range of the GZ of 6 and 46 week old mice was significantly different from 4 week old mice (MD = − 25.60 for 6 weeks and MD = − 26.17 for 46 weeks), whereas no significant difference was noted between 6 and 46 weeks (MD = − 0.56). MR did not significantly decrease, though variance in the percentage of epithelium occupied led to the doubling of standard deviation in mature lenses.

The development of the low density CZ occurs between postnatal weeks 4 and 6 in mice. During this transition, the density slope minimum moves to *θ* = 75° and remains stable through to 46 weeks. In contrast, the density slope maximum moves from *θ* = 95° to *θ* = 90° (Fig. 3A) between 4 and 6 weeks. These characteristics remain stable throughout the phase of rapid lens volume growth, in contrast with their placement during the major transition event from spheroid to lentoid lens shape between 4 and 6 weeks.

### Meridional row length decays with increasing age

The LECs in the MR are highly organized in cell columns^11^. They occupy between 4 and 6% of the total epithelium throughout the murine lifespan. Young animals show tightly packed MR, with individual columns offset to one another by approximately half a cell width and little variance in their number and distribution. In contrast, mature animals demonstrate a wider variance in the percentage of lens epithelium covered by the MR (Fig. 3B) consistent with reports highlighting the age-related decay in MR organization^11^.

## Discussion

Here we present a novel, rapid method to determine important eye lens metrics, such as the shape, aspect ratio and cell density in whole lenses (see the “Methods” and Supplementary section for full details of the methods, the processing pipeline and analyses). It confirms the previously reported age-dependent changes in cell density, particularly those in the GZ and MR of the epithelium of lenses taken from C57/BL6J mice. The method reduces time and resources needed for analysis, and eliminates data distortion encountered in previous methods such as flat mounting the epithelium^25–27^, which is a problem for small, round animal lenses such as those in the mouse eye^11^, and serially sectioning the lens^28,29^ or physically restraining with potential to deform lens shape^18^. Moreover, the algorithm independently calculates the position of the anterior pole and delivers cell density measurements using a three-dimensional polar coordinate system to position every cell in both *xyz* and *spherical angle* format. Subsequent statistical analyses identify differences in zonal density and cell location for lenses across an age range (C57/BL6J 4–46 weeks). This is particularly important to characterize the effects of the many mutations that affect either lens size or the epithelial cell distribution and their effect on the ageing tissue.

In this study we have characterized a previously unreported stage in postnatal lens development, namely the transition of the lens from a spheroid to lentoid shape coinciding with the development of the CZ to GZ density gradient. This change is not adequately accounted for in previous models^11,14,18^ and in so doing diminished the potential functional impact of this shape change on the role of the lens in establishing the refractive properties of the eye^7^. It also adds to known changes in the refractive properties of the C57/BL6J eye when emmetropia is established between 4 and 6 weeks. We additionally evidence the importance of standardizing the measurement of lens metrics and expanding on their integration with geometric eye properties. Creating a fully integrated map of molecular, cellular, and geometric lens characteristics will enhance our understanding of refraction in the eye and will allow the exploration of the mechanistic detail that leads, for example,  to myopia^30^.

Previous reports have tracked the total LEC number across the lifespan of the C57/BL6J mouse^11,14^. The total number of LECs reaches a peak of approximately 50,000 by 4 weeks, followed by a decline to about 43,000 by postnatal week 12^14^. This number then remains stable over the rest of the lifespan. Using just two variables (cell proliferation rate and cell surface area) an elegant model of lens growth for the C57/BL6J mouse was produced. The observed decline in cell number between postnatal weeks 4 and 12 is not accompanied by a stutter in or stalling of lens growth, as the rate of differentiation temporarily exceeds the rate of proliferation following this fourth postnatal week, but goes on to reach an equilibrium at 12 weeks^14^. A model applicable to the epithelium of all vertebrate species was based also on just two parameters, one being the proliferation rate ratio of the peripheral and central regions and the second, a dimensionless pull-through parameter to account for the transition and exit of epithelial cells into the lens body as LFCs^11^. Both models have advanced lens research and been important hypothesis-generating tools, but both used cell proliferation molecular markers as a key model parameter which could limit their utility and application to mouse mutants with altered lens growth and proliferation rates. The method presented here efficiently measures key lens parameters, and allows for the first time the opportunity to correlate changes in epithelial cell distribution and lens shape changes with the development of emmetropia in the mouse eye that are not accounted for in the two models of lens growth.

Emmetropia is the regulation of ocular growth to ensure that a focused image falls on the retina^21^. In many mammals, including the mouse, this is established in the postnatal period between weeks 4 and 6 in the C57/BL6J strain^7,22^ when the lens is in its most active growth phase^14^. The mouse has only recently been accepted as a model to study emmetropia due to differences observed between C57/BL6J and other inbred strains^7,21,30^. Practical issues for measuring refraction in such a small eye also had to be overcome in order to monitor emmetropia in the mouse eye^5,21,22,31^. It is well documented that there are significant differences in eye size between the different mouse strains^23^, but it has not yet been investigated whether the organization and density of the cells in the lens epithelium of these different strains is the same or different. Eye refraction is also highly variable between genetic backgrounds, with the 129SvJ strain identified as having among the largest refractive errors and the C57/BL6J strain the least^5,30^. Recent investigations have identified gene candidates in retinal signaling pathways that are linked to myopia in mice and humans^30^. Whilst many of the identified pathways are also very pertinent to the lens, it is worth noting in particular that the actin cytoskeleton, ephrin signaling, and glutathione biochemistry^30^ were amongst those identified as important pathways contributing to refractive eye development.

**Figure 4:**
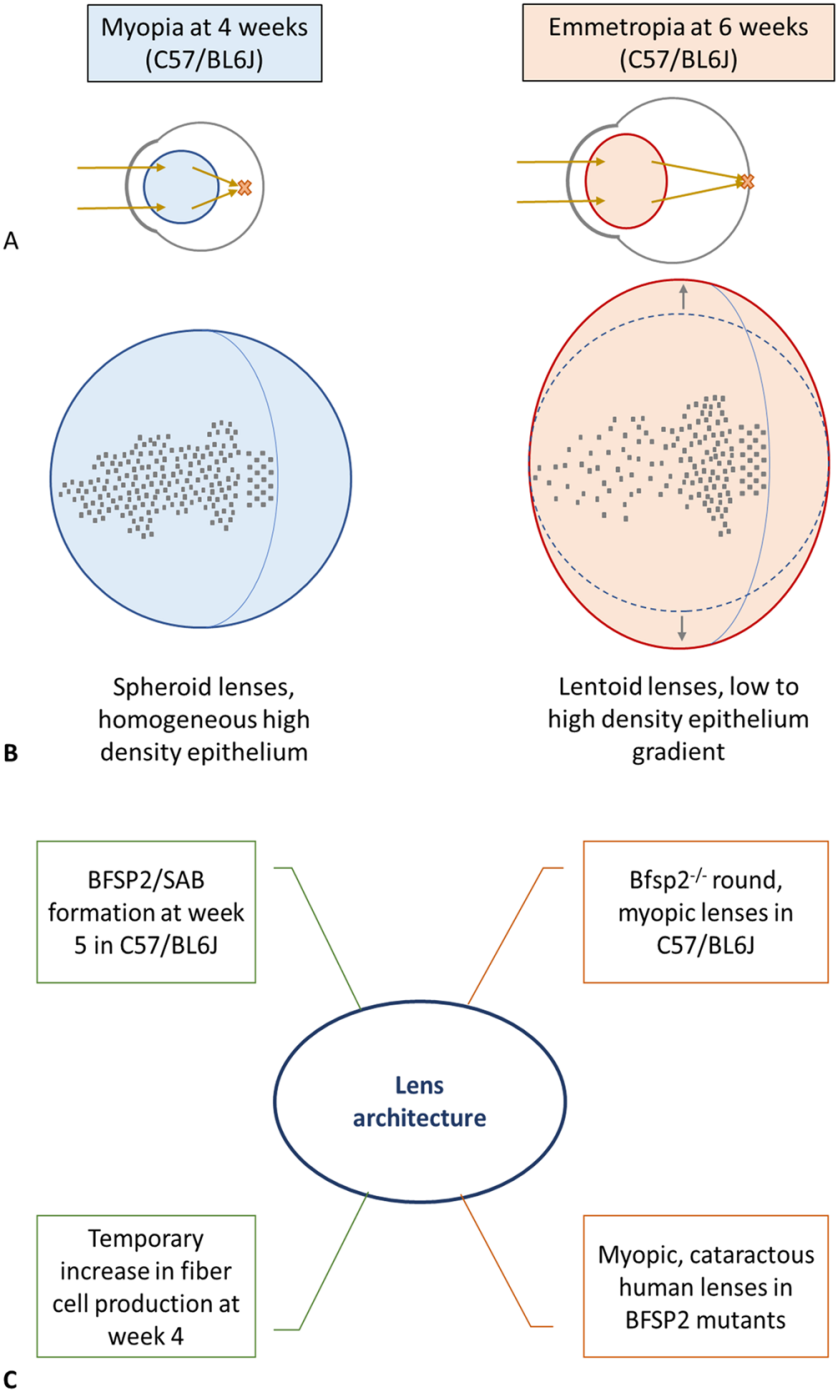
Key events for a functional mouse lens. In the eye, the lens refracts light onto the retina, as seen in (**A**). This function fully develops in mice, and specifically the C57/BL6J strain, by postnatal week 6 with the complete establishment of emmetropia. At postnatal week 4, the eye is myopic and images are not focused on the retina; rather they are formed in front of the retina as indicated by the orange X in (**A**). In this study we have measured both a change in lens shape from spheroid to lentoid from postnatal week 4 to week 6, as illustrated in (**B**), and observed a concomitant change in the lens epithelial cell distribution. These cellular and anatomical changes correlate with the development of emmetropia^7, 21^ by postnatal week 6 and the reported formation of the strain specific, cytoskeletal structure called the SAB^26,35^ at postnatal week 5. The absence of the cytoskeletal protein Bfsp2 has been correlated with both refractive error and the loss of SABs. Mutations in *BFSP2* are linked to inherited high myopia and cataract in humans, as summarized in (**C**).

There is good reason to consider lens defects as contributing to refractive error in conditions such as myopia. Mutations in the lens specific intermediate filament protein Bfp2 caused cataract in a family first noted for their very high myopia^32^. The optical properties of the mouse lens are altered in the *Bfsp*2 knockout and *Bfsp2* mutations present in FvB and 129SvJ, but not C57/BL6J mouse strains^33,34^. The 129SvJ strain also presents with very significant refractive error^30^. Links between the development of emmetropia and the lenticular cytoskeleton have also been identified in C57/BL6J mice with the formation of polygonal actin networks called sequestered actin bundles (SABs) at postnatal day 35^35^. These SABs were subsequently shown to be integrated with the intermediate filament protein Bfsp2 and are absent when Bfsp2 is also absent^36^, although the timing of this integration was not recorded. When the *Bfsp2* knockout was introduced onto the C57/BL6J genetic background, the lens retained a more spheroid than lentoid shape compared to wild type littermates^37^. Our characterization of the major transitional event between weeks 4 and 6 sheds new light on the correlation between the development of emmetropia and the effect of cytoskeletal elements during the most active growth phase of the lens. The dramatic change from spheroid to lentoid shape coupled with the development of the mature LEC density gradient clearly illustrate how molecular processes could affect optical function for the lens (Fig. 4).

The transition from spheroid to lentoid shape between the fourth and sixth postnatal week is a significant lens remodeling event which coincides with changes in the optical power of lenses in C57/BL6J mice^7,22^. One study identified that a shape change occurred between week 5 and 12 for lenses of the C57/BL6J strain^37^, but it was unclear from those data whether this change occurred when or after emmetropia is established in this strain of mouse^7^. It is known that the varying concentration of β- and γ-crystallins contribute to the development of the refractive gradient index, successfully maintaining lens function in periods of rapid growth^14,38^. The increased radial deposition of fiber cells during this time period^14^ can be explained to a certain extent by the hypothesis that the lens grows to occupy its greater ocular environment. Coupled with the presence of growth factor gradients, which promote proliferation only in equatorial, high LEC density regions^19^, this shape change can be highlighted as a natural consequence of epithelial organization. However, camera eye-type lenses are seen in a variety of spheroid and lentoid shapes. The spherical lenses of mollusks^39^, cephalopods^40^, and teleosts^41,42^ offer a stark contrast to mammal and avian lentoid lenses^43–45^. Refractive plasticity studies demonstrated the relationship between introduced refractive aberrations and physiological vision input during development in avian^46,47^ and primate lenses^47,48^, further hinting at the contribution of early optical signals to the establishment of normal lens architecture. Epithelial organization in mammals follows the low to high density gradient distribution with relatively short MR (Fig. 1). Producing accurate three-dimensional maps of lens epithelia is an invaluable tool especially when then paired with comprehensive omic datasets such as iSyte (https://research.bioinformatics.udel.edu/iSyTE/ppi/).

## Method

### Lens collection and processing

Four week old, 6 week old, 16 week old, and 46 week old C57/BL6J mice (n = 16) were euthanized by cervical dislocation according to the UK Animals (Scientific Procedures) Act 1986. Ethical approval for these studies was given by the Animal Welfare and Ethical Review Committee, University of Durham. All animals were fed *ad libitum* and received standard care including social housing at constant temperature. Whole eyes were removed, and a small v-shaped incision made at the optic nerve head to facilitate overnight fixation in 4% (w/v) paraformaldehyde (Sigma, Germany). The eyes were then washed in PBS and stored in PBS containing 0.5% (w/v) paraformaldehyde. The zonules remained intact until lenses were removed from the fixed eye using a previously established protocol^49^ and then permeabilized with 0.5% (v/v) Triton X-100 (Sigma, Germany) PBS for twenty minutes at room temperature and then stained with 10 μM Hoechst 33342 (Sigma, Germany) for thirty minutes at room temperature. Eyes and lenses were stored in PBS containing 0.5% (w/v) paraformaldehyde until imaging. Technical guidance on the identification and mitigation of dissection artefacts are given in the Supplementary File Section 1.

### Whole lens mounting

**Figure 5:**
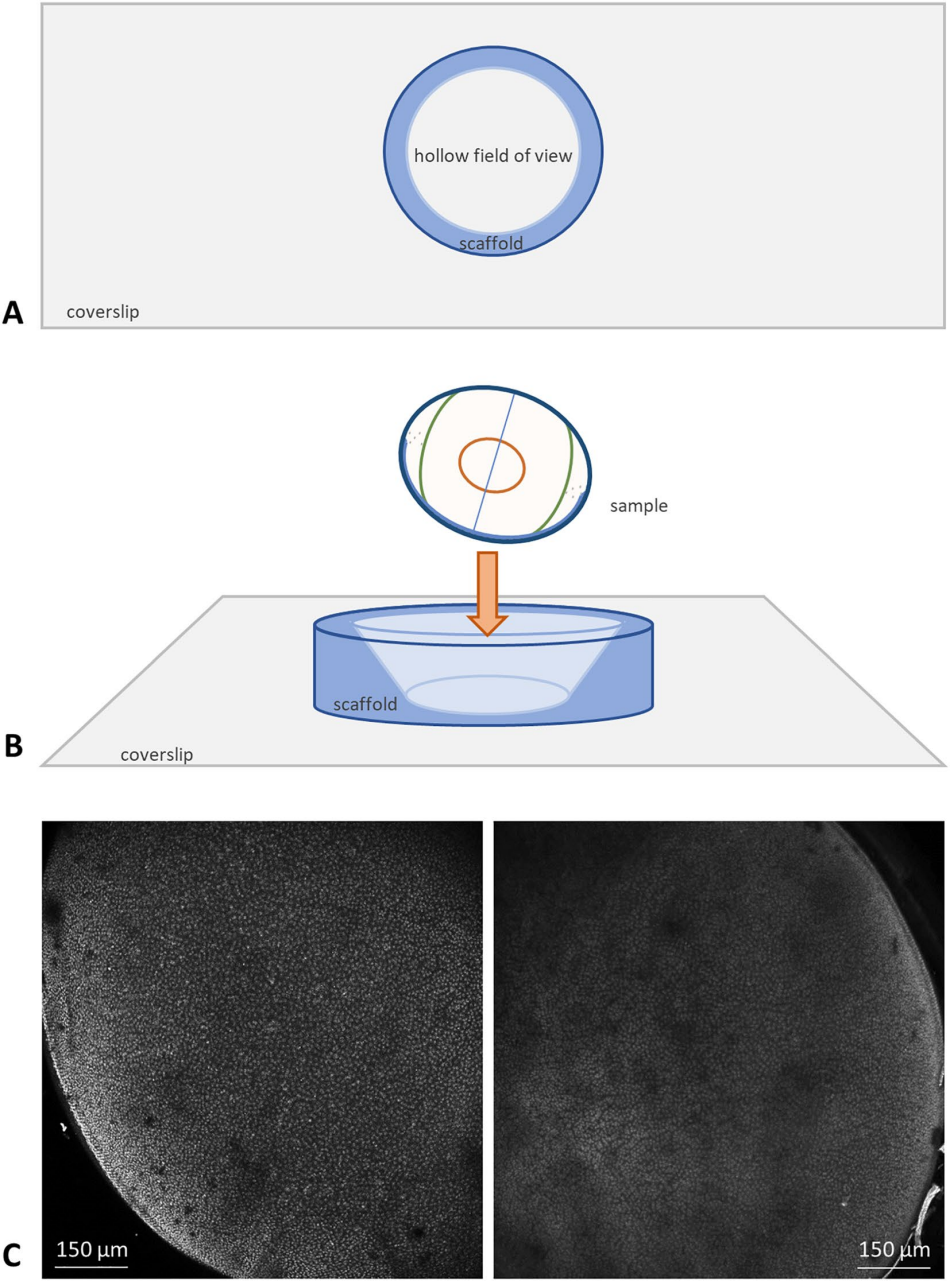
Schematic representation of lens mounting sample data capture (maximum intensity projections). (**A**,**B**) fixed and stained lenses are placed in a hollow polymerized acrylamide matrix on a glass coverslip and imaged immersed in an appropriate mounting medium. Exported data can be reconstructed into a two-dimensional z-stack, as seen in (**C**). Shown here are two murine lenses, C57/BL6J at 46 weeks (left panel) and 16 weeks (right panel) stained with Hoechst 33342. Scale bars 150 μm.

Whole lenses were mounted at an approximately 35° angle in an in-house system consisting of glass coverslips and hollow polyacrylamide cones, as seen in Fig. 5. Polyacrylamide is optically transparent and chemically inert and therefore does not interfere with the data capture process whilst providing a stable scaffold for each lens sample.

### Data capture

Images were recorded using a Leica SP5 II confocal microscope equipped with an HXC APO × 10/0.40 NA oil (n = 1.518) immersion objective. Data were collected using 100 Hz/line scan speed with a 2-line average and bidirectional scanning using a 355 nm laser line (3rd harmonic of a NdYAG laser) with 2mW laser power. The microscope was equipped with a triple channel imaging detector comprising of two conventional PMT systems and a HyD hybrid avalanche photodiode detector. Frame size was determined at 1024 × 1024 pixel, with a 1 Airy disc unit pinhole diameter and z-step size of 5.98 μm. The voxel size was determined as 1515.2 nm × 1515.2 nm × 5977.2 nm (xyz). Nuclear Hoechst staining presented in grey.

### Data reconstruction

Three-dimensional reconstruction of z-stacks was performed in Fiji^50,51^. Data analysis was performed in MatLab (MATLAB, 2018. *9*.*7*.*0*.*1190202* (R2019b), Natick, Massachusetts: The MathWorks Inc.) using an in-house developed software package (https://github.com/BoguslawObara/eye-lense3d).

### In silico analysis

#### Nuclei detection

**Figure 6:**
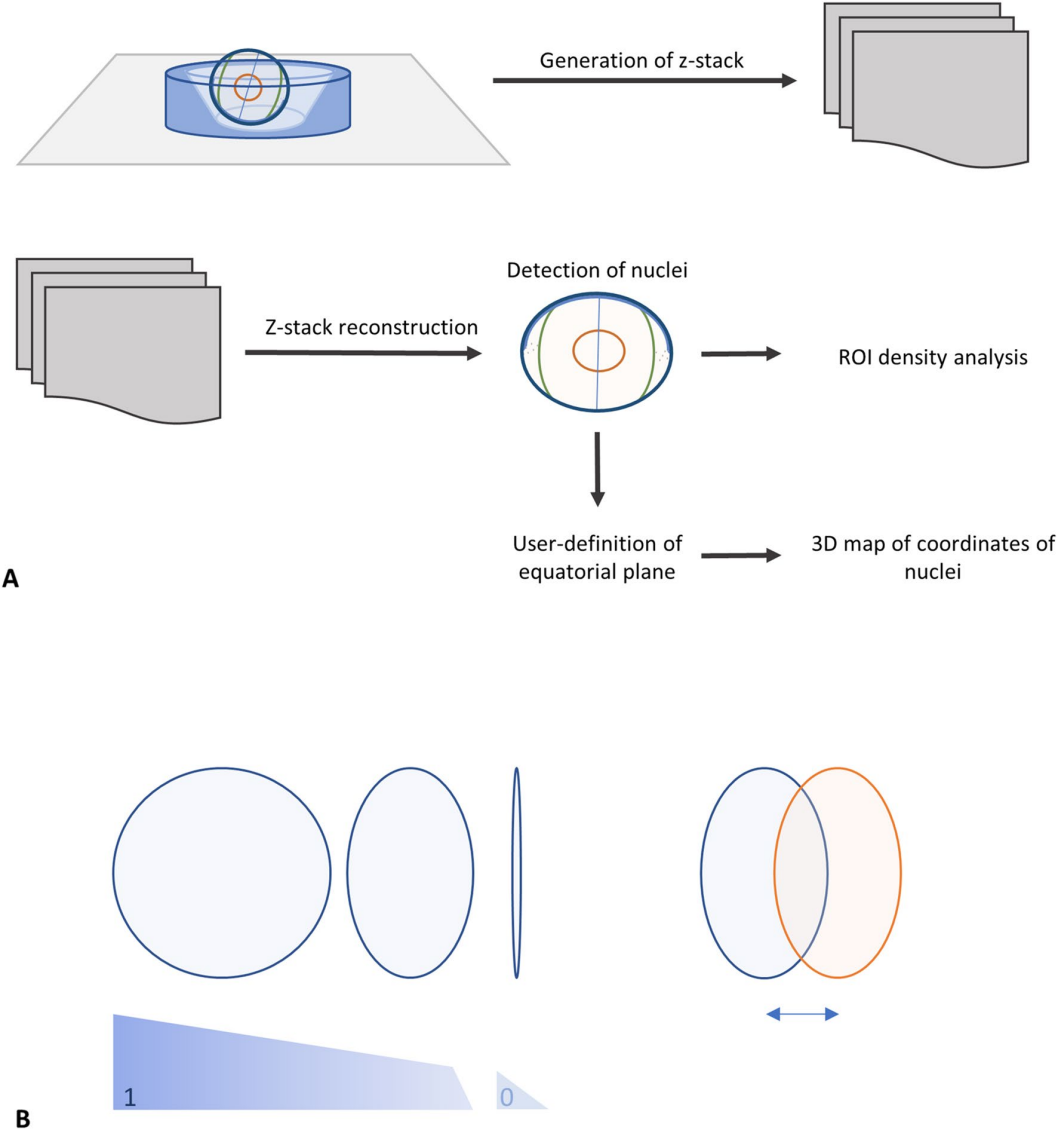
Analysis pipeline and LEC nuclei definition. (**A**) Processing pipeline, from sample imaging to 3D map generation. A z-stack is captured for each lens sample and reconstructed prior to the detection of LEC nuclei. Density analysis of ROIs can be performed as shown in Fig. 8A, and 3D coordinate maps of detected nuclei are produced following the user-definition of an equatorial plane and the subsequent geometrical definition of the anterior pole. (**B**) LEC nucleus detection is based on user-defined circularity and thresholding values. Image quality appropriate thresholding are required for successful watershed segmentation of partially overlapping nuclei.

The processing pipeline is summarized in Fig. 6A. To automatically detect the locations of 3D nuclei, a 3D input image is convolved with a Laplacian of Gaussian kernel defined by the radius *R*. Such image convolution results in a 3D likelihood map of the local maxima in the image. The locations of the local maxima are then estimated by 3D grayscale morphological dilation of the likelihood map. The image map dilation involves assigning to each pixel, the maximum value found over the neighborhood defined by the spherical structural element or a radius *R*. Nuclear circularity and intensity thresholding are user-defined values, as seen in Fig. 6B, to account for differences in image quality. Further details on nuclei detection and the elimination of user bias can be found in Supplementary File Sections 2 and 3.

#### Region of interest analysis

**Figure 7:**
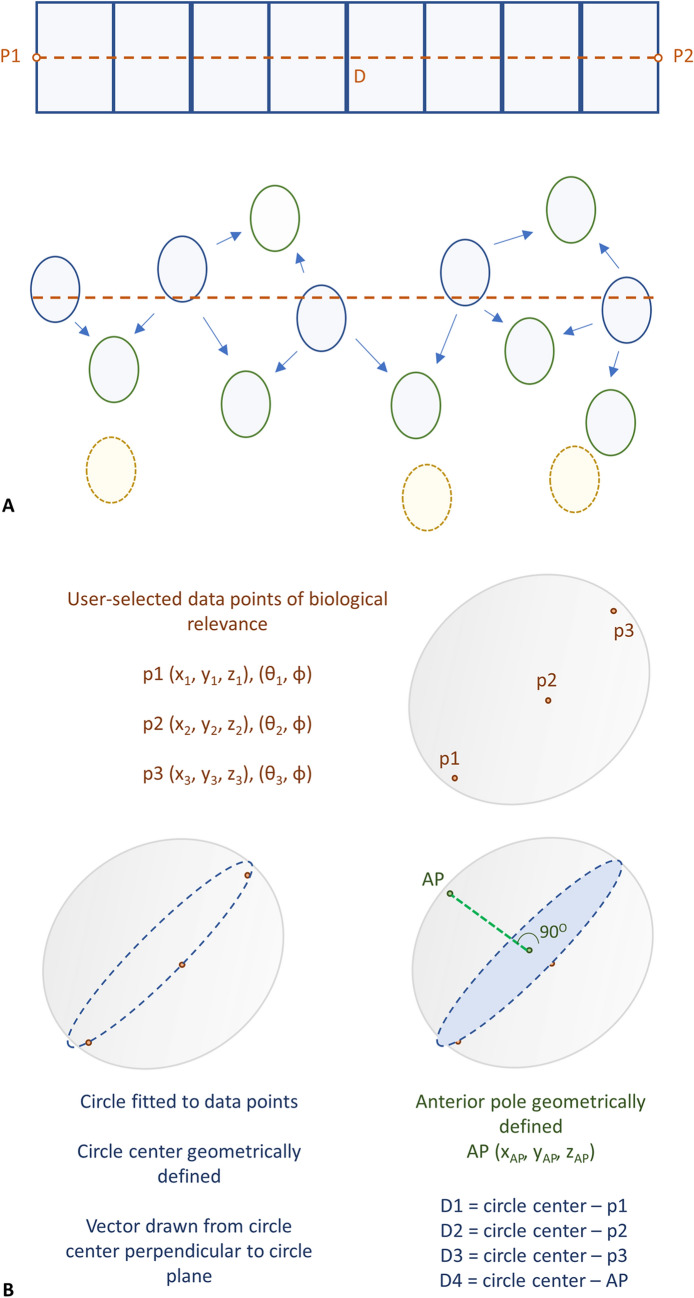
Region of interest analysis and LEC nuclei global coordinate assignment. (**A**) A user-defined set of two points (P1, P2) form a vector which traverses the epithelium from CZ to MR. A rectangular grid divided into equal segments annotates the region of interest, and LEC nuclei in contact with the central vector are used as anchoring points to determine their direct neighbouring nuclei. The distance D is calculated from the image metadata, and LEC nuclear density is calculated as nuclei per neighbourhood/μM. (**B**) Three user-defined points (p1, p2, p3) are selected with equal *φ* at the boundary between the GZ and the MR, and the point coordinates are extracted from the image metadata. A circle is fitted to these three points and is used to define a near-equatorial plane bisecting the lens. A vector is produced from the center of the circle perpendicular to the near-equatorial plane and is projected towards the hemisphere with detected nuclei. The point where the vector meets a detected LEC nucleus is determined as the anterior pole (AP).

**Figure 8:**
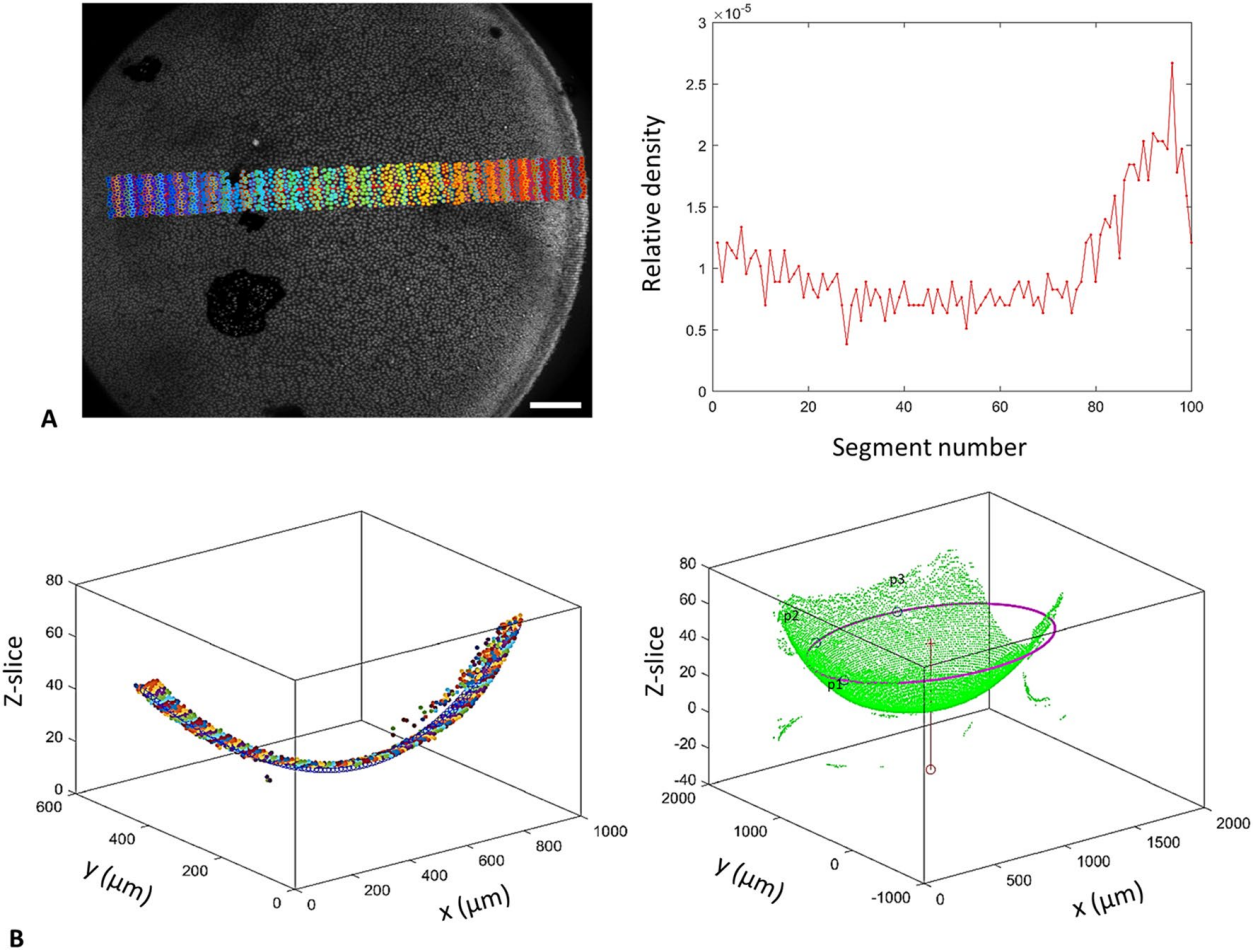
ROI analysis and LEC nuclei global coordinate assignment. (**A**) ROI analysis output: the maximum intensity guide image with the color-annotated grid and segmented nuclei is produced in parallel to the LEC nuclear density graph. (**B**) ROI and global coordinate assignment output: the segmented nuclei from the ROI grid are reconstructed in 3D according to the image metadata. All detected nuclei, the user-defined points (p1, p2, p3), the near-equatorial circle plane and the perpendicular vector are also reconstructed in 3D.

Two user-selected start and end points (*P1, P2*) and their distance *D*, are used to define a rectangular region of interest (ROI) which is divided into equal length grids. These grids are used to estimate LEC nuclei’s local area density, which is defined as the number of nuclei traversed by the vector (*P1, P2)* and their immediate neighbors in each grid (Fig. 7). Nuclear count is then normalized as nuclei per neighborhood/μm as seen in Fig. 8A. ROI analysis can be used as a high throughput method to characterise changes in LEC density across large sample numbers due to the lack of variance in *φ*, as described in Supplementary File Sect. 5, and due to the multiple sample loading function of the software.

#### Global coordinate assignment

Three user-defined points are selected at the boundary between the germinative region and the meridional rows (*p1, p2, p3*) and their coordinates are extracted from the image metadata. A best-fit circle is defined using these three points, and a near-equatorial plane is established. Using the center of the circle, a vector is drawn perpendicular to the defined plane and is extended to the data-populated direction. The cross section of this vector and the data is geometrically defined as the anterior pole (AP) of the lens. Distances *D1, D2, D3* and *D4* are calculated between points *p1, p2, p3, AP* and the center of the fitted circle to generate radial length data, as seen in Fig. 7. Global coordinates are assigned to all detected lens nuclei in *xyz* format.

#### Alignment of datasets and rotation of detected nuclei

Due to the inevitable variability in the placement of lenses in the support matrix seen in Fig. 5 and 6, data must be rotated prior to the assignment of spherical angle coordinates for detected nuclei. A series or rotation matrices (available in Supplementary File Sect. 4) are used to realign the collected data, with the equatorial region defined as *θ* = 0 and *θ* = 2π. Global spherical angle coordinates are then assigned for all detected nuclei (Fig. 8B).

All data are extracted in tabulated form for downstream statistical analysis. One-way ANOVA with α = 0.05 was used to determine the effect of age on zonal density and zonal borders across age-grouped subject data. Post hoc analysis was performed using Tukey HSD.

## Conclusion

Temporal 3D mapping of cell populations is a powerful tool in development and ageing studies. Overcoming the barrier of user-friendly in silico analysis could maximize output from large datasets. Our processing pipeline presents a computational strategy to map cell density and its distribution on the surface of a spheroid, taking advantage of the orientating features and regular shape of the eye lens.

We have generated user-friendly LEC maps which retain global lens geometry, improving upon the efficiency and accessibility of previous techniques aimed at modelling lens epithelia. Our novel mounting method and in silico analysis package have reduced data acquisition and processing time tenfold as compared to previous methods whilst also creating a platform to allow the integration of expression markers and their lens distribution. Further, we have reported and characterized a major event in the development of emmetropia in the mouse eye, which is consistent with the formation of LEC zonal separation and a lens shape change at a key postnatal period in mouse lens growth.

## Supplementary information

Supplementary Information

## Abbreviations

LSCM: Laser scanning confocal microscope
LEC: Lens epithelial cell
LFC: Lens fiber cell
CZ: Central zone
GZ: Germinative zone
MR: Meridional rows
FGF: Fibroblast growth factor
AP: Anterior pole
SAB: Sequestered actin bundle
MD: Mean difference
ROI: Region of interest
HSD: Honest significant difference
