# Supplementary material for: Three-dimensional data capture and analysis of intact eye lenses evidences emmetropia-associated changes in epithelial cell organization
Source: Sci Rep. 2020 Oct 9;10:16898. doi: 10.1038/s41598-020-73625-9 (PMC7547080; doi:10.1038/s41598-020-73625-9)
Supplement: Supplementary file 1 — Supplementary Information [file 41598_2020_73625_MOESM1_ESM.docx]

**Three-dimensional Data Capture and Analysis of Intact Eye Lenses evidences Emmetropia-associated Changes in Epithelial Cell Organization.**

Alexia A. Kalligeraki^1^, Archie Isted^1^, Miguel Jarrin^1^, Alice Uwineza^1^, Robert Pal^2^, Chris Saunter^3^, John Girkin^3^, Boguslaw Obara^4*^, Roy A Quinlan^1*^

*^1^Department of Biosciences, ^2^Department of Chemistry, ^3^Department of Physics, ^4^Department of Computer Science, Durham University, South Road Science Site, Durham DH1 3LE, UK*

*Corresponding authors: e-mail: [boguslaw.obara@durham.ac.uk](mailto:boguslaw.obara@durham.ac.uk); r.a.quinlan@durham.ac.uk

**SUPPLEMENTARY APPENDIX**

1. **Parameters for successful imaging**

Appropriate sample preparation is essential for the successful production of LEC nuclei maps. We have identified three major areas for sample quality control:

1. Sample integrity.
2. Stain contrast.
3. Microscopy parameters.

The preservation of sample integrity is essential to lens preparation. Lenses must be thoroughly fixed at room temperature to avoid the formation of “cold cataracts”, which are then mostly irreversible in dissected eyes. Careful dissection of each specimen must be considered, to avoid injuring the epithelium either during the removal of the lens from the eye or during the removal of residual iris tissue adherent to the lens. We recommend dissection is undertaken in filtered neutral buffered solutions, such as PBS, to avoid contamination of the lens from airborne fibers and microplastics that are readily attracted to the lens capsule, as seen in Supplementary Figure S1. Examples of injured and contaminated lenses follow.


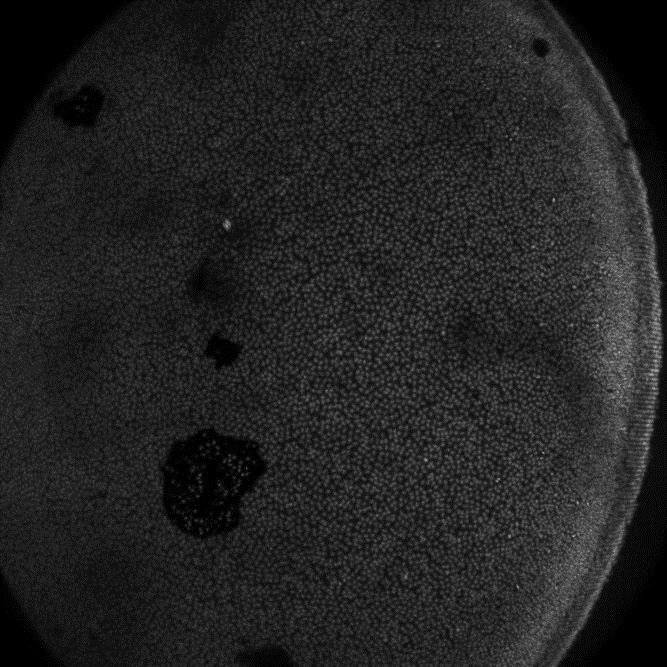

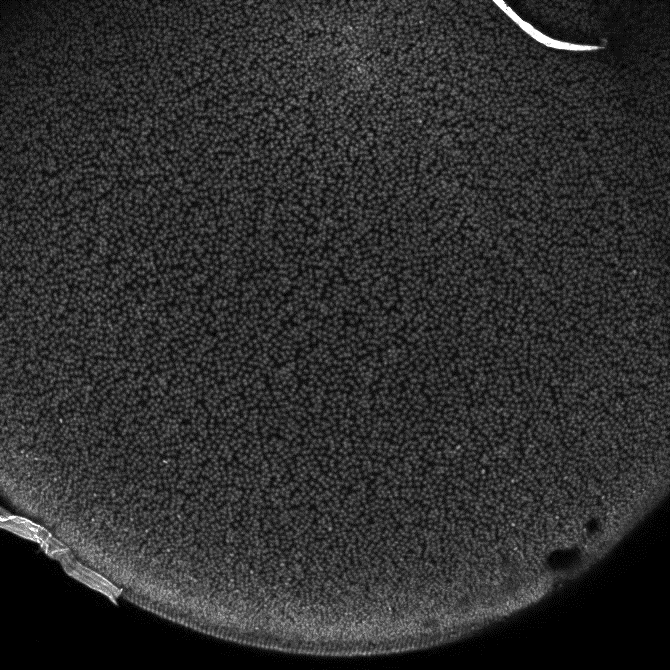


Supplementary Figure S1. Common preparative injuries and contaminants to the lens epithelium. Dissection can lead to the retention of tissue from the iris (left panel above, circled area 1) or puncture sites with the loss of epithelial cells (left panel above, circled area 2; right panel above, circled area 4). Dissection and processing without the lens being immersed in aqueous solutions can lead to contamination from airborne fibers of variable size and source (right panel above, circled area, 3). Both are maximum projection images of 6-week C57/BL6J murine lenses, vitally stained with Hoechst 33342. Scale bar 200 μm.

Thorough fixation is paramount to ensure good signal to noise for the vital dyes to be used and for minimum background fluorescence in stained samples. The lens capsule can be a barrier to the penetration of chemical dyes or antibody-mediated staining, and so some permeabilization is required. We have successfully used a range of non-ionic surfactants such as Triton X-100, Brij-35 and polysorbitol 20 (Tween 20) to overcome this issue. Digestion of the lens capsule with collagenase is incompatible with this preparation method, as over-digestion compromises the integrity of the lens epithelium.

Finally, we have used standardized data capture parameters across each dataset as the analysis directly involves measurement of physical characteristics such as radial distance. In this study we have used a standard image resolution for all data captured (1024x1024 pixels) and have oversampled by 10% on both axial tails through a smaller z-section height (5.98μm) to ensure the fluorescence intensity remains constant throughout the z-stack. As our processing suite relies on voxel size to generate measurements in physical distance units, consistent pixel size (image resolution) and depth (z-stack height) are strongly recommended to avoid user error during analysis.

1. **Data input for cell detection**

As mentioned previously, we have used a standard voxel size for all samples processed to ensure physical distances are accurately calculated for map reconstruction. The computational identification of individual cell nuclei is based on minimum thresholding of both their size and fluorescence intensity. The user-defined parameters to execute this function and to ensure successful watershed segmentation of “figure of eight” nuclei (i.e. two nuclei where boundaries appear to touch or overlap) are termed “sphericity” and “thresholding”. Sphericity values should be between 0.1 and 1, with 0 representing a line and 1 a perfect circle, and the thresholding value of fluorescence intensity should be defined based on the quality of the signal to noise ratio of each stained sample.

1. **The aim to eliminate user bias in sample collection**

The placement and orientation of each lens in the polyacrylamide matrix is difficult to control precisely and so the orientation of the epithelium relative to the microscope objective will vary between samples. To counter this, the processing software incorporates a rotation function (see Section 4 below) prior to allocating coordinates for each LEC nucleus data point. This function allowed the user to select their preferred orientation and therefore maximize dataset consistency.

A 3D point cloud is initially generated and shows the data as-captured, namely in the orientation as viewed by the microscope objective. These data can then be rotated on the three principal axes to generate consistent maps of the LECs. In the first instance, a user-defined reference point is selected for *xy* rotation. We found the GZ-MR boundary to be the most reliable, biologically relevant, and easy to identify feature as it lies on the same *φ* in each lens. Since the lens epithelium is a monolayer of cells, this eliminates the need for z definition during the *xy* rotation. Following this first rotation, the user-defined equator is aligned in z to *θ =0* and *θ =2π.*

A circle is fitted to the data on the equatorial plane, and an orthogonal vector is projected from its center. The single data point (i.e. nucleus) with the least squared distance is designated as the anterior pole, as seen in Figure 8. Data points are then assigned *xyz* and *θφ* coordinates and are exported in txt format for analysis outside the processing suite.

Maintaining the balance between user input and the mathematical determination of relevant geometric characteristics requires some initial training and appreciation for the biological system under investigation. An experienced user is more efficient at distinguishing biological features in the lens epithelium. We have chosen to limit user input to easily described, and therefore unequivocally identified, biological features of the lens such as the boundary between the GZ and the MR. Once identified, simple geometry is used to procure downstream data as to minimise user bias as far as possible.

1. **Data rotation**

The rotation matrices used to reorientate lens datasets are as follows.

The boundary between the GZ and MR in the equatorial region of the lens was set to *θ =0* and *θ =2π*. Rotations around each axis are performed by the following matrices:

| $R_{x}\left( \theta\right)=\left[ \begin{matrix} 1 & 0 & 0 \\ 0 & \cos\theta& -\sin\theta\\ 0 & \sin\theta& \cos\theta\end{matrix} \right]$ | (1) |
| --- | --- |
| $R_{y}\left( \theta\right)=\left[ \begin{matrix} \cos\theta& 0 & \sin\theta\\ 0 & 1 & 0 \\ -\sin\theta& 0 & \cos\theta\end{matrix} \right]$ | (2) |
| $R_{z} \left( \theta\right)=\left[ \begin{matrix} \cos\theta& -\sin\theta& 0 \\ \sin\theta& \cos\theta& 0 \\ 0 & 0 & 1 \end{matrix} \right]$ | (3) |

These rotations are performed counter-clockwise by a *θ* angle around each relative axis, with inversion of the sign producing clockwise rotation [1]. The data are thus aligned to a user-defined plane of biological significance, in this instance the equatorial plane with the selected GZ-MR boundary parallel to the x-axis. The user-defined vector which marks the GZ-MR boundary has an *xyz* alignment derived from the initial placement of the lens in the polyacrylamide matrix. To rotate this vector in the *xy* plane, the following matrix is given:

| $\left[ \begin{matrix} 1 & 0 & 0 \\ 0 & A & -B \\ 0 & B & A \end{matrix} \right]\left[ \begin{aligned} x \\ y \\ z \end{aligned} \right]=\left[ \begin{aligned} x \\ yA-zB \\ yB+zA \end{aligned} \right]=\left[ \begin{aligned} u \\ v \\ t \end{aligned} \right]$ | (4) |
| --- | --- |

Where *A* and *B* are functions to be determined, and *u* and *v* are the new (x,y) coordinates of the vector. The equations can be solved for *A* and *B*:

| $\begin{aligned} x=u \\ yA-zB=v \\ yB+zA=0 \end{aligned}$ | (5) |
| --- | --- |
| $\sqrt{x^{2}+y^{2}+z^{2}}=\sqrt{u^{2}+v^{2}}$ | (6) |
| $\begin{aligned} A=\frac{y}{d} \\ B=\frac{-z}{d} \end{aligned}$ | (7) |
| $d=\sqrt{y^{2}+z^{2}}$ | (8) |

These can then be adapted to the rotation matrix for the *xy* plane as follows:

| $R_{xy}=\left[ \begin{matrix} 1 & 0 & 0 \\ 0 & \frac{y}{d} & \frac{z}{d} \\ 0 & \frac{-z}{d} & \frac{y}{d} \end{matrix} \right]$ | (9) |
| --- | --- |

From matrices 1 and 9, we can calculate the *θ* angle of the vector rotation, and then complete the whole datapoint rotation using these and matrices 2 and 3.

| $\cos\theta=\frac{y}{d}$  $\sin\theta=\frac{z}{d}$ | (10) |
| --- | --- |

1. **Determining LEC nuclei distribution patterns across *θ* and *φ***

The nuclear distribution across *θ* for lens epithelium has previously been characterised [2–4], with a low to high density gradient observed from anterior pole to equator being a typical profile for a mammalian lens. We examined LEC nuclear distribution for *φ* in C57/BL6J lenses to assess whether epithelial radii have distinct nuclear density patterns at different *θ*.

Density averages of *θ* were calculated for each *φ*, with no discernible pattern being apparent at postnatal weeks 4, 6, 46, and 112 as seen in Supplementary Figure S2.


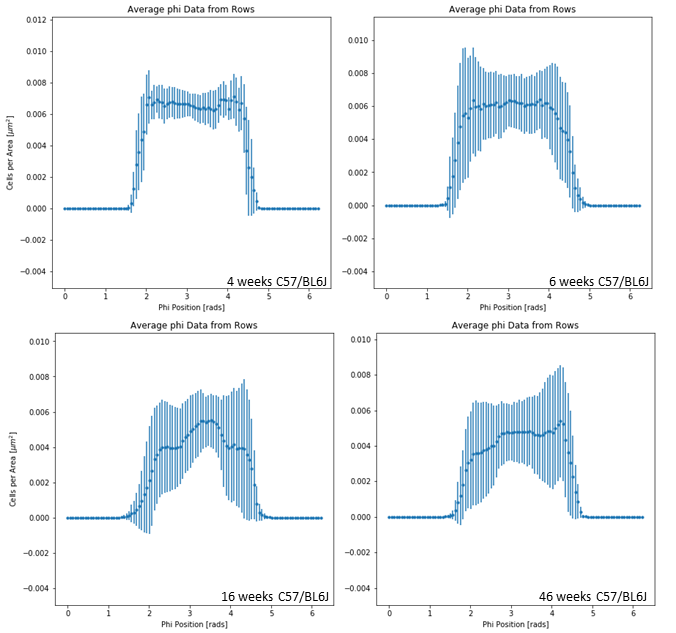


Supplementary Figure S2. Density averages of all *θ* values for each given *φ*. C57/BL6J mice at 4 weeks and 6 weeks show no pattern of density in *φ*. At 16 weeks a small peak emerges at the anterior pole, but otherwise no pattern can be seen. This peak is lost by 46 weeks. *Χ^2^* fits revealed straight lines within error for all data sets. n = 16.

Separate analysis of the CZ and GZ revealed a homogeneous distribution of LEC nuclei at *θ* for each given *φ*, confirming the overall findings from Supplementary Figure S2. As seen in Supplementary Figure S3, the overall error is decreased when LEC nuclei are sampled in two different zones and *Χ^2^* analysis supported these findings.


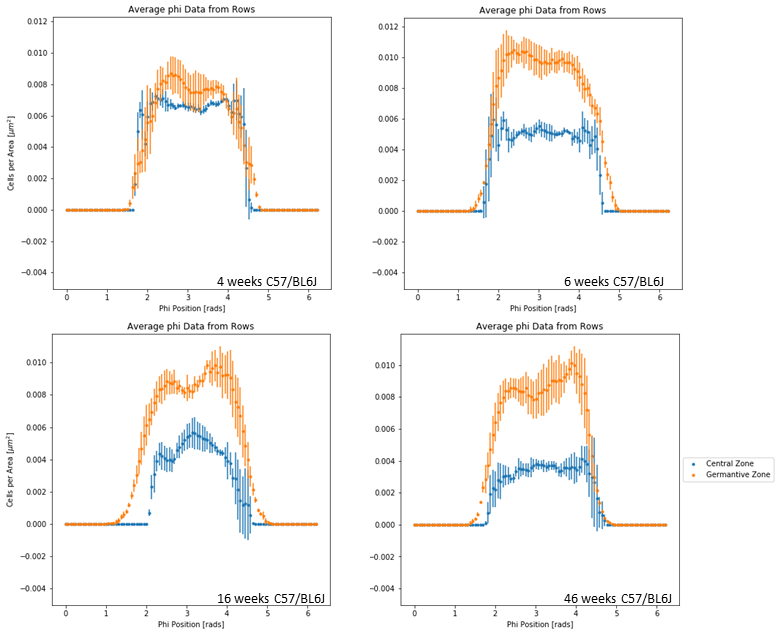


Supplementary Figure S3. Density averages of all *θ* values, at the GZ and CZ specifically, for each given *φ*. As in Supplementary Figure 2 the distributions shown here also confirm no pattern for either the GZ or CZ regions. *Χ^2^* fits confirmed this, evidencing no patterns in *φ*, either over the entire lens or localized to a single region. n = 16.

Due to these observations, ROI analyses of lens epithelium regions can be safely extrapolated for the entire lens at each *φ*. A “scalloping” effect has previously been observed in radiation-exposed murine lenses [5] altering their aspect ratio, which could be attributed to persistent localised changes in LEC density. Our whole tissue 3D imaging and mapping technique allows further analysis of similar effects and localised LEC responses to environmental stressors such as radiation, opening the opportunity for much more extensive data mining from limited sample numbers.

1. **Data normalisation in Region of Interest analyses**

Region of interest (ROI) analyses provide a rapid tool to process large sample datasets using only the initial steps of the processing software. Since the distribution of LECs across *φ* at any given *θ* has no distinct patterning, as shown in Supplementary Figure S2, any cell density normalisation needs to be performed across a polar to equatorial vector to account for different lens sizes and changes in curvature.

A three-dimensional ROI analysis requires two steps. Initially, nuclear density would be calculated on *xy* to establish cell distribution in each z-slice. As z length is equal between measurements, z-slices would be assigned as individual sequential segments in the density histogram seen in Figure 8. However, this method has the following complications and limitations.

From a data capture perspective, it is impossible to place different lenses in the containment matrix in identical positions during the imaging process. As the objective is a fixed data capture source, any variance in sample angle will be reflected in z slice absolute densities. Further, the optimal z-slice length for an objective needed to achieve the necessary field of view is approximately 6 μm resulting in partial overlap of nuclei across consecutive z segments. It is necessary then to assign a minimum area threshold to nuclei to sort them into appropriate z segments.

We also need to recognize that the biological regions of the lens epithelium (central zone, germinative zone, meridional rows) do not align with individual z-slices because of the angle required to image each sample and produce the broadest field of view. Consider the z-slices captured from near-equatorial latitudes relative to the microscope objective. Due to the ellipsoidal shape of adult mouse lenses, z-slices furthest from the microscope objective capture cells from all three biologically relevant regions from the lens epithelium, namely the central and germinative zones, and the meridional rows. As all three regions can be characterized by unique density profiles, normalization of absolute density measurements within z-slices would not only be erroneous due to the incorrect segmentation of nuclei but would also introduce variance that would render the dataset unusable from a statistical analysis perspective.

Taking the above into account, it is important to highlight that the lens epithelium is a cuboidal cell monolayer, and as such there is no requirement to consider three-dimensional data normalization for ROI analyses.

Lens epithelium density can be calculated for the curvature of the lens itself using two-dimensional area segmentation. A user-defined rectangular ROI is fitted to the lens surface, which is then segmented into equal and appropriately sized sequential regions. Nuclei within ROI boundaries are used to produce an absolute density histogram, where x is segment number and y is nuclei/segment. Normalization across x length is trivial for the comparison of different sized lenses, as the physical z length of the ROI can be calculated from the image metadata using the known voxel size. Area-based measurements are still imperfect impeding further analysis. As radial length increases towards the equator, a static y chord length produces a decreasing arc length (L) resulting in oversampling of near anterior polar areas of the CZ and under sampling of equatorial areas of the GZ as seen in Supplementary Figure S4. To account for this, the static length y would have to be assigned to the arc defining each segment. Though this might seem trivial, the production of a static arc parameter requires the establishment of polar coordinates for each detected nucleus which then renders the ROI analysis irrelevant, as it produces an absolute density map for all captured nuclei.


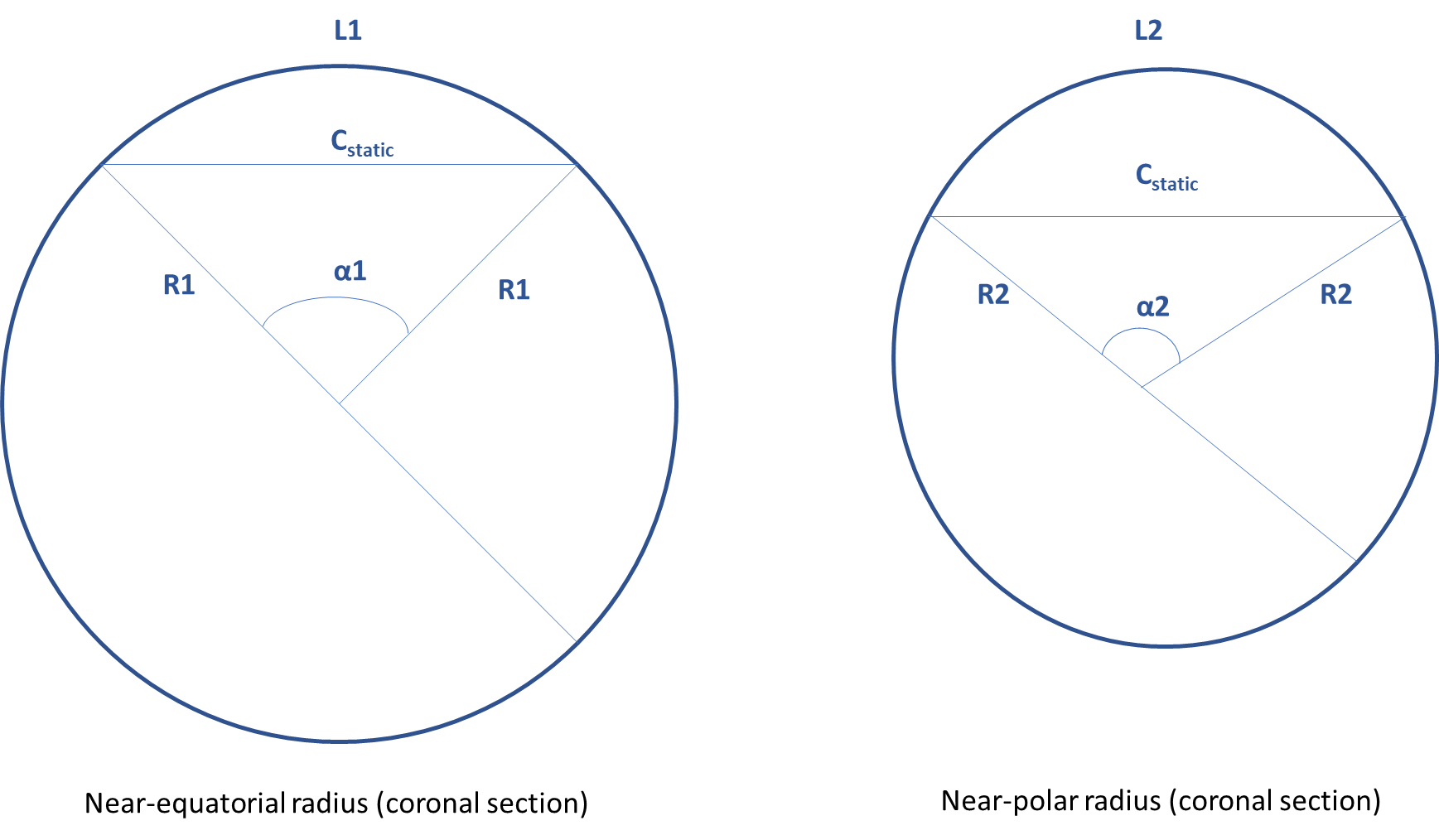


Supplementary Figure S4. A static chord length (C_static_) defining each ROI segment produces a variable arc length (L) at different lens *φ* angles.

Since we have shown there is no discernible density gradient in *φ* across each lens *θ*, density measurements can be simplified as a vector-based analysis. Nuclei in contact with the central vector of the user-defined ROI are used as anchoring point for a nearest neighbor analysis; “nearest neighbor” are nuclei directly adjacent to central vector nuclei. These data are then divided into arbitrary static length segments. The total length of the central vector is easily calculated from image metadata voxel size as mentioned previously, therefore cell density is normalized to (number nuclei in each neighborhood)/μm for each individual segment to produce density histograms from the anterior pole in the CZ to the MR.

As data are now normalized to (nuclei per neighborhood)/μm in each segment, direct comparisons can be made between size-matched lenses in terms of absolute cell number per μm.

1. Arfken, G. B., Weber, H. J. & Harris, F. E. Chapter 2 - Determinants and Matrices. in (eds. Arfken, G. B., Weber, H. J. & Harris, F. E. B. T.-M. M. for P. (Seventh E.) 83–121 (Academic Press, 2013). doi:https://doi.org/10.1016/B978-0-12-384654-9.00002-5.

2. Šikić, H., Shi, Y., Lubura, S. & Bassnett, S. A stochastic model of eye lens growth. *J. Theor. Biol.* **376**, 15–31 (2015).

3. Bassnett, S. & Šikić, H. The lens growth process. *Prog. Retin. Eye Res.* **60**, 181–200 (2017).

4. Wu, J. J. *et al.* A dimensionless ordered pull-through model of the mammalian lens epithelium evidences scaling across species and explains the age-dependent changes in cell density in the human lens. *J. R. Soc. Interface* **12**, (2015).

5. Markiewicz, E. *et al.* Nonlinear ionizing radiation-induced changes in eye lens cell proliferation, cyclin D1 expression and lens shape. *Open Biol.* **5**, 150011 (2015).
